# Finger pressing task data collected with and without post-trial performance feedback

**DOI:** 10.1016/j.dib.2020.105127

**Published:** 2020-01-11

**Authors:** S. Balamurugan, S.K.M. Varadhan

**Affiliations:** Department of Applied Mechanics, Indian Institute of Technology Madras, Chennai, India

**Keywords:** Finger pressing task, MVC, Feedback, Epilogue

## Abstract

The dataset presented in the article consists of finger forces of participants during a finger pressing task. The finger pressing task involves the production of fingertip forces using Index, Middle, Ring, and Little (I, M, R&L) fingers of the right hand. The participant performed two types of task, namely MVC task and visual occlusion task. The participants completed the Maximum Voluntary Contraction (MVC) task first, where they were instructed to produce maximum possible force from each finger individually and all fingers together. The visually occluded finger pressing task followed the MVC task. In this task, the participant's visual feedback was removed after 8s. There were two conditions in this task, one with post-trial performance feedback (referred to as “epilogue” condition in this manuscript) and another that does not have this post-trial performance feedback (referred to as “no epilogue” condition in this manuscript). The epilogue condition is a particular case of post-trial visual feedback where, at the end of each trial, the performance in that trial is shown to the participant. This was followed by the next trial. Normalization of force levels for visual occlusion tasks was performed for the forces with the participants produced in the MVC task. Fourteen healthy participants were recruited for performing the experiments. For the experiments, they were instructed to produce fingertip forces using four fingers of the right hand with the target line at 15% MVC (15% of the force that they produced in the MVC task). The two visual occlusion conditions had 30 trials each. In both conditions, a single trial lasted 16 s. For the initial 8 s, there is visual feedback, which follows an eight-second visual occlusion period where there is no visual feedback. The dataset consists of three files; the first file has the data of Maximum Voluntary Contraction (MVC) data, the second file has the data for the “without epilogue” condition, and the third file has the data of “epilogue” case.

**Table:** Specifications Table

Subject	Behavioral Neuroscience
Specific subject area	Motor control
Type of data	Fingertip pressing force data
How data were acquired	The data was acquired using PCB force sensors (PCB Piezotronics INC, NY, USA) and Nano 17 force/torque sensors (ATI Industrial Automation, Garner NC, USA). Customized software was developed using LabVIEW to collect the data from the sensors at 200Hz.
Data format	Raw data in CSV format, MAT files. Raw data of the entire experiment is provided.
Parameters for data collection	The data has three files for different conditions. The first file has MVC data, the second file contains data for without epilogue case, and the third file contains data for with epilogue case. For all three cases, experiments performed were different. Hence fingertip forces and conditions can be considered as two factors.
Description of data collection	Participants performed fingertip pressing tasks on force sensors through the course of the experiments. For the MVC task, each participant performed ten trials. The MVC task is followed by the visual occlusion task that has two conditions, without epilogue and with the epilogue. They performed 30 trials each for both the cases. This experiment was performed to explore the learning achieved in the finger force production task when provided with the epilogue. Direct URL to data: https://doi.org/10.17632/7d8rm729z4.2
Data source location	Institution: Indian Institution of Technology Madras City: Chennai Country: India
Data accessibility	Repository name: Mendeley Data Data identification number: 10.17632/7d8rm729z4.2 Direct URL to data: https://doi.org/10.17632/7d8rm729z4.2
Related research article	Preprint: Balamurugan S, Dhanush Rachaveti, and Varadhan SKM (Corresponding Author), Role of post-trial visual feedback on unintentional force drift during isometric finger force production tasks, bioRxiv, https://doi.org/10.1101/864413

**Table:** 

**Value of the Data** • The data can help in understanding how fingertip forces change during two conditions, i.e., with and without post-trial visual feedback (epilogue). • The dataset presented in the article can help people working in fields such as finger mechanics, neural control & modeling, and cognitive aspects of learning, such as memory consolidation [[1], [2], [3], [4], [5], [6], [7], [8]]. • The data is valuable since it provides feedback of fingertip force produced by all the four fingers along with a particular case of post-trial visual feedback, i.e., epilogue. • The data can also be used to understand the spectral characteristics of fingertip forces [[9], [10], [11]] in the two conditions.

## 1 Data description

The dataset presented in the article comprises three files namely MVC task, Without epilogue task and With epilogue task. The first file is a CSV file that contains Maximum Voluntary Contraction (MVC) forces of Index, Middle, Ring, Little fingers individually and all the four fingers together for each participant. The dimension of the file is 15 rows x 6 columns, where the first row of the file gives details about the participant ID and finger used for force production. The first column of the file represents the Participant ID, the second column represents the data of MVC force from the index finger, the third column represents data from the middle finger, the fourth column represents data from the ring finger, and the fifth column represents data from the little finger. The sixth column represents the MVC force when all four fingers produce force and the representation of data of the MVC task is shown in Table 1. The data in the MVC task data represents the force in Newton, and sample data can be seen in Table 2.

**Table 1:** Represents the data arrangement for the MVC task performed by all subjects. It has 6 columns and 14 rows in total.

Column Number	Representation of the column
Column number 1	Represents participant ID
Column number 2	Represents MVC force of the index finger
Column number 3	Represents MVC force of the middle finger
Column number 4	Represents MVC force of the ring finger
Column number 5	Represents MVC force of the little finger
Column number 6	Represents MVC force of all fingers together

**Table 2:** Represents the sample data in MVC task data.

Sample Data	Representation of the data
35.46	Represents the force produced by the participant in Newton
13.45	Represents the force produced by the participant in Newton

**Fig. 1:**
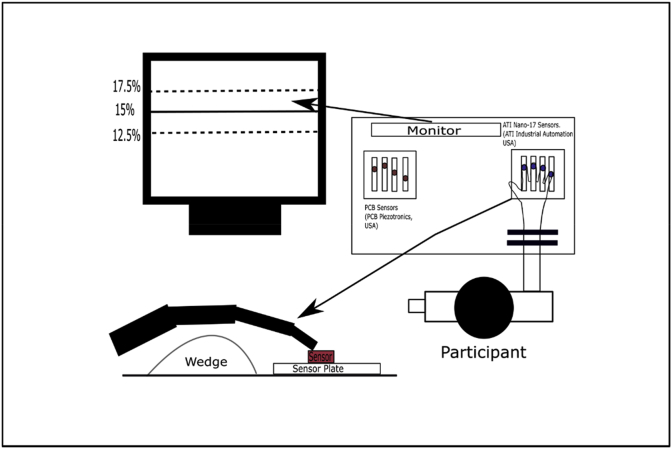
**Schematic diagram of the experimental setup.** The experimental setup used in the experiment consists of two different sets of sensors for each of the four digits (I-Index, M-Middle, R-Ring, and L-Little). These sets of sensors were PCB 208C02 single-axis strain gauge sensors and Nano 17, six-axis strain gauge force sensors. Both the sensor sets were fixed to their respective tables separately. The strain gauge sensors were used in a Maximum Voluntary Contraction (MVC) task to measure the maximum value of force produced by each finger. The Nano 17 sensors were used to measure forces in the visual occlusion experiment. The participant's palm rested on a wedge after restraining the forearm and wrist movement with Velcro straps. Participants were instructed to maintain the normal isometric forces of I, M, R, and L fingers over the solid horizontal line between the boundaries of the dotted lines.

**Fig. 2:**
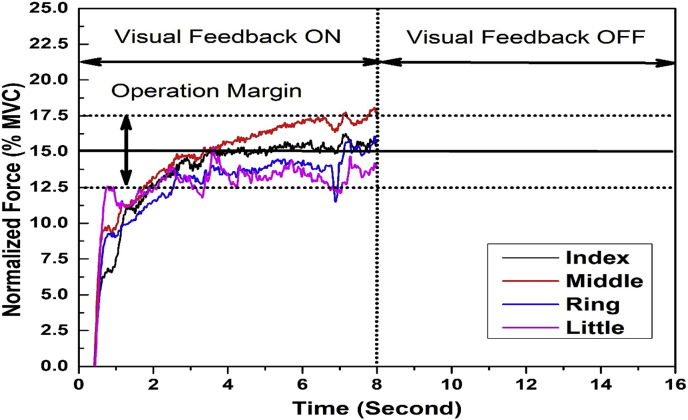
**The Visual representation of feedback shown during a trial in both visual occlusion tasks.** The feedback shown to the participants consists of a solid horizontal line representing 15% of their MVC, computed during the MVC task with 12.5% and 17.5% MVCs as the operating margin. The performance of the fingertip forces between the operating margins is considered to be acceptable. The total duration of the task is 16 seconds with visual feedback ON for the first 8 s, followed by the visual feedback OFF for the final 8 seconds of the task. The color lines indicate the forces produced by the respective fingers I, M, R, and L. This online feedback shown to the participants remains the same for both, with the epilogue and without the epilogue conditions. Y-axis represents the isometric force produced by each finger normalized for their respective MVCs, while the X-axis shows the total duration of the task.

**Fig. 3:**
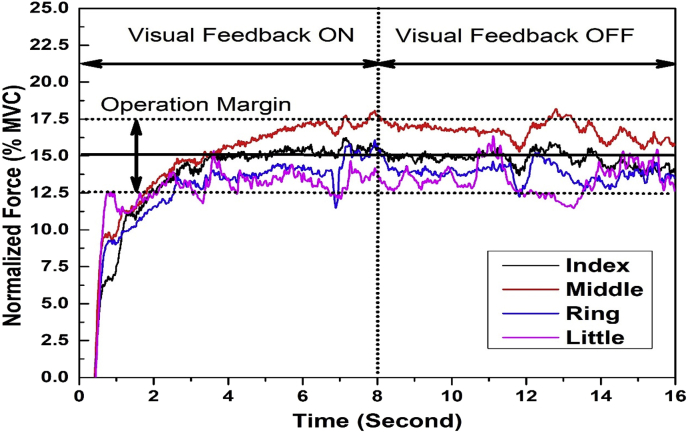
**The Visual representation shown to the participant after each trial in the visual occlusion task with the epilogue.** The feedback shown to participants at the end of each trial in the condition involving epilogue. The total duration of the task is 16 seconds with visual feedback ON for the first 8 s, followed by the visual feedback OFF for the final 8 seconds of the task. The difference between the epilogue and the non-epilogue condition is that the epilogue condition involves post-trial feedback of the just-concluded trial, while for non-epilogue condition, there is no post-trial feedback. The post-trial feedback was shown for 29 trials (excluding the first trial). The color lines indicate the forces produced by the respective fingers I, M, R, and L. Y-axis represent the isometric force produced by each finger normalized for their respective MVCs, while the X-axis shows the total duration of the task.

The dimension of the without epilogue task (second file) and with epilogue task (third file) is 14 cell × 30 cell .MAT file. MAT extension files are the binary data container format that the Matlab program uses and was developed by Mathworks. MAT files are categorized as data files that can include variables, functions, arrays and other information. MAT files can be saved in a variety of formats as long as users choose to save them in a version that belongs to the MATLAB Preferences. However, the.MAT file can also be opened using open-source platforms such as Python and GNU Octave. They have data of 30 trials (rows) performed by all 14 subjects (column) for the with and without epilogue conditions. Each column represents data from one participant. For example, column one corresponds to participant one; column two corresponds to participant two, and so on. Each row corresponds to a trial number, i.e., the 28th row has the data of participants corresponding to the 28th trial. Each cell of the second and third file has 4 columns with the columns one to four representing the force data from the index, middle, ring, and little fingers respectively. Each column had 3200 rows that correspond to data collected for 16 seconds sampled at 200 Hz (16 × 200 = 3200 data points). Like the MVC task data, the data of the visual occlusion task is also measured in Newton. The data file is organized within the root folder named with the type of task performed, namely MVC task, without epilogue task, and with epilogue task. The experimental apparatus is illustrated in Fig. 1. The visual representation of a single complete trial (as seen by the participant during the trial) is shown in Fig. 2. The representation of the post-trial epilogue is shown in Fig. 3. The details of the experiment containing trial duration, number of trials are shown in Table 3.

**Table 3:** Details about the tasks, number of trials and duration of each trial.

Sl. No.	Task name	Duration of each trial (Second)	No. of trials
1	MVC task	10	10
2	Visual occlusion task without the epilogue	16	30
3	Visual occlusion task with the epilogue	16	30

## 2 Experimental design, materials, and methods

### 2.1 Statement of ethics and recruitment of participants

Fourteen healthy right-hand dominant participants (8 male and 6 female, mean ± standard deviation: 23 ± 2.96 years) volunteered to participate in the experiment. The experiments excluded participants with any history of musculoskeletal or neurological illness. The participants were right handed according to self-reported hand choice for writing and eating. Institutional ethics committee of IIT Madras approved the experimental procedures (Approval number: **IEC/2016/02/VSK-8/18.** Full name of the committee that granted the approval: Institutional ethics committee of Indian Institute of Technology Madras). All experimental sessions followed the procedures approved by the Institutional ethics committee of IIT Madras. All participants provided written informed consent before the start of the experiment. All participants were naive to the purpose of the experiment. Participants were provided INR 500 as compensation for their time and effort. An experimental session lasted approximately 1.5–2 hours.

### 2.2 Experimental setup

The apparatus used in the experiment consists of two different sets of sensors, PCB 208C02 sensors (PCB Piezotronics INC, NY, USA) with amplifiers 482C05 and Nano 17 sensors (ATI Industrial Automation, NC, USA). Each sensor set has four sensors each, for four digits (I-Index, M-Middle, R-Ring, and L-Little). Both the sensor sets were fixed to their respective tables separately. PCB sensors were used for the Maximum Voluntary Contraction (MVC) task, where the maximum value of force produced by each of the fingers individually and all fingers collectively were measured. The Nano 17 sensors were used to measure force in the visual occlusion experiment that requires measurement of forces with considerably lower magnitude. A wooden wedge was placed under the participant's palm for supporting the hand. The participant's forearm and the wrist were fixed to the table using Velcro straps before the commencement of the experiment. The diameter of each of the ATI Nano 17 sensor is 17 mm, and the PCB 208C02 sensor is 15.88 mm. The center to center distance of the four sensors was 25 mm. Sensor position was adjusted in the anterior-posterior direction depending on participant's finger length. 100-grit sandpaper was used on the contact surface of all sensors to increase the friction between the digits and sensors. Participants received visual feedback of the applied load force of all fingers (Index, Middle, Ring, and Little) on a 17-inch LCD monitor placed at a distance of approximately 75cm. Participants were seated comfortably in a chair with their right forearm resting on top of the table. They were asked to extend the fingers of their right hand and place the fleshy part of the distal phalanges facing downwards on the set of sensors, as shown in Fig. 1. Participants were instructed to keep their wrist in contact with the table, and their arm was wrapped with Velcro to avoid unwanted force production from the upper arm till the end of the experiment. All four fingers were placed on the designated sensor, and the participants were informed to alert the experimenter if they were facing discomfort at any point during the experimental period. Participants were given a briefing about the experiment before signing the informed consent form.

### 2.3 Experimental tasks

#### 2.3.1 MVC task

MVC task is used to find the maximum force that can be produced by each finger individually and collectively. In this task, the participant needs to perform 10 trials (2 with each finger individually and 2 using all the fingers collectively) with maximum voluntary contractions (MVC) to produce a maximal force that they could produce using the finger as instructed to them. The trials were performed in a sequence with 2 minutes of the rest interval. For every participant, the maximum force produced from the two trials is considered as the individual finger's MVC and is used for normalization in further experimentation.

#### 2.3.2 Visual occlusion task without the epilogue

In this task, participants were asked to match the force line at 15% of MVC by keeping their force values of fingers between the outer two dotted lines at 12.5 and 17.5% MVC. The total task duration was 16 seconds. After 8 seconds from the start of a trial, visual feedback was removed, and participants were instructed to produce finger forces with no visual feedback till the end of the trial, i.e., till 16th second. In the visual occlusion period, participants were instructed to try and maintain the force at the same level as they were producing just before the start of occlusion. The visual representation of a single complete trial in this task is shown in Fig. 2. Participants performed 30 trials in this condition.

#### 2.3.3 Visual occlusion task with the epilogue

This task is similar to the previous task, except that the participants will be provided with the epilogue of the recently concluded trial, before the start of the next trial. However, during experimentation, the visual feedback shown to the participant is the same as the previous task. The representation of the post-trial epilogue is shown in Fig. 3. This epilogue can be used to make corrections in the next trial. Participants performed 30 trials in total, with epilogue given for 29 trials (excluding the first trial). A 5-min break was enforced between the visual occlusion tasks. Additional breaks were given if participants requested.

#### 2.3.4 Software (LabVIEW) control

A customized code in LabVIEW was used to collect the data for the finger pressing task using two different sets of sensors, PCB sensors (PCB Piezotronics INC, NY, USA) and Nano 17 sensors (ATI Industrial Automation, Garner NC, USA). An experimental trial lasts for 16 seconds, and the LabVIEW code collects the data of all fingertip forces during the complete course of the trial.

#### 2.3.5 Experimental protocol

All participants completed the experiments in a single session with breaks in between the three tasks as well as individual trials. The MVC task involves 10 trials (2 trials for each finger individually and 2 trials for all fingers together) where the participants were instructed to produce the maximum possible force that can be produced by them. For each finger and all finger together, only the trial that has the maximum MVC force is considered and used for further experimentation. The MVC task is followed by the visual occlusion task, which involves force production using the index, middle, ring, and little finger together. Each participant has to produce 15% of the force that they produced during the MVC task for the visual occlusion task. This is performed for 60 trials (30 trials for without epilogue case and 30 trials for with epilogue case). The epilogue is a particular case of post-trial feedback, where the final outcome of the just-concluded trial is shown to the participants. For experiments without the epilogue, no post-trial feedback is shown to the participants, whereas for experiments involving epilogue, the epilogue of the just-concluded trial is shown. The details of the experiment containing trial duration, number of trials are shown in Table 3.
